# Feed Supplementation with Red Seaweeds, *Chondrus crispus* and *Sarcodiotheca gaudichaudii*, Reduce *Salmonella* Enteritidis in Laying Hens

**DOI:** 10.3389/fmicb.2017.00567

**Published:** 2017-04-10

**Authors:** Garima Kulshreshtha, Bruce Rathgeber, Janice MacIsaac, Martine Boulianne, Lehoux Brigitte, Glenn Stratton, Nikhil A. Thomas, Alan T. Critchley, Jeff Hafting, Balakrishnan Prithiviraj

**Affiliations:** ^1^Department of Plant, Food, and Environmental Sciences, Faculty of Agriculture, Dalhousie University, TruroNS, Canada; ^2^Department of Animal Science and Aquaculture, Faculty of Agriculture, Dalhousie University, TruroNS, Canada; ^3^Atlantic Poultry Research Institute, Dalhousie University, TruroNS, Canada; ^4^Faculté de Médecine Vétérinaire, Université de Montréal, Saint-HyacintheQC, Canada; ^5^Department of Microbiology and Immunology, Faculty of Medicine, Dalhousie University, HalifaxNS, Canada; ^6^Acadian Seaplants Limited, DartmouthNS, Canada

**Keywords:** *Chondrus crispus*, Sarcodiotheca gaudichaudii, Salmonella Enteritidis, antibiotics, layer hens

## Abstract

*Salmonella* Enteritidis is vertically transmitted to eggs from laying hens through infected ovaries and oviducts. *S.* Enteritidis can also penetrate the eggshell from contaminated feces. Reducing *S.* Enteritidis in laying hens is vital to provide safer eggs and minimize the spread of salmonellosis to humans. Antibiotics have been widely used to control bacterial diseases in broilers and laying hens. However, there is a major concern that the use of antibiotics leads to the development of antibiotic resistance and adverse effects on microbiota of the treated birds. Thus, there is an interest in developing alternatives to antibiotics, such as dietary prebiotics. In the present study, feed supplemented with the red seaweeds: *Chondrus crispus* (CC) or *Sarcodiotheca gaudichaudii* (SG), was offered to laying hens late in production to control *S.* Enteritidis. Diets contained one of the following; 2% or 4% *Chondrus crispus* (CC2, and CC4, respectively) or *Sarcodiotheca gaudichaudii* (SG2 and SG4, respectively). Chlortetracycline was used in the positive control diet. During week-4, 48 birds were orally challenged with 2 × 10^9^ CFU/mL of *S.* Enteritidis. Eggs and fecal samples were collected 1, 3, 5, and 7 days’ post inoculation. Birds were euthanized and organs (ceca, ovary, liver, and spleen) were sampled and analyzed for the presence of *S.* Enteritidis, 7 days’ post inoculation. Results showed that seaweed reduced the negative effect on body weight and egg production in *S.* Enteritidis-challenged laying hens. Analysis of fecal samples showed that the antibiotic (CTC) reduced *S.* Enteritidis in the intestinal tract and fecal samples, 3 days’ post inoculation. Fecal samples from Chlortetracycline and CC4 supplemented birds tested negative for *S.* Enteritidis on days 5 and 7 post inoculation (lowest detection limit = 10^-1^). *S.* Enteritidis colonization in the ceca was also significantly reduced in birds fed CC (4%) and Chlortetracycline. Blood serum profiles revealed that there were no significant differences in serum aspartate aminotransferase (AST) and sodium. However, the level of serum immunoglobulin (IgA) was higher in the CC4 treatment. The relative abundance of *Lactobacillus acidophilus* was significantly higher in CC4 while, the abundance of the pathogenic bacteria, *Clostridium perfringens* and *Salmonella* Enteritidis were reduced compared to control. Results indicate that feed supplemented with 4% CC is effective in providing protection against *Salmonella* Enteritidis colonization in laying hens.

## Introduction

*Salmonella* *enterica* serovar Enteritidis (*S*. Enteritidis) is a major cause of egg-associated salmonellosis in humans (Guard-Petter, 2001). Despite the ongoing implementation of several control and prevention measures, the number of *S*. Enteritidis cases has increased since 2005, making it the most common serotype responsible for *Salmonella* infections in Canada (NESP) (Center for Disease Control and Prevention [CDC], 2012). Major concerns associated with *Salmonella* infection in poultry include loss in productivity, higher mortality, and contamination of egg-associated poultry products consumed by humans (Chai et al., 2012). Therefore, due to adverse effects on economics and human health, the safety of poultry products is a priority for the government, poultry producers and the consumers. Chicken acts as a reservoir host for *S*. Enteritidis infection, and this pathogen is carried into the human food chain through consumption of contaminated, raw, or undercooked poultry products (Guard-Petter, 2001). Previous epidemiological studies have confirmed the relation between human salmonellosis and the consumption of poultry products (FDA, 2009; Center for Disease Control and Prevention [CDC], 2012).

*Salmonella* can enter poultry through the oral fecal route and colonizes the gastrointestinal tract. This leads to shedding of *Salmonella* in excrement for several weeks without any detectable clinical symptoms in the infected bird. Moreover, *S.* Enteritidis can contaminate an egg from inside and on the outer surface of eggshell. Contamination from inside can occur through the direct transmission of *Salmonella* into egg contents resulting in its proliferation in the yolk, albumen, eggshell membranes, or eggshells before oviposition. Moreover, an egg can harbor *Salmonella* by horizontal transmission through the eggshell from the colonized gut or infected feces during or after oviposition (Gantois et al., 2008). Over the past 50 years, antibiotics have been used as growth promoters to enhance the production and to prevent diseases (Barrow et al., 1987***;*** Berg, 1995). However, sub therapeutic use of antibiotics in poultry has been under scientific and public scrutiny. The use of antibiotics in poultry has been linked to an increase in antibiotic resistant strains of bacteria, which pose a serious threat to the effective treatment of bacterial infections and human diseases. There is an increasing interest from poultry producers to find alternatives to antibiotics (Baurhoo et al., 2009).

The immune system provides innate protection against pathogen infection. The first line of defense against *S*. Enteritidis infection is provided by the intestinal mucosal immune system including mucosal immunoglobulin A (IgA) and mucosa associated lymphocytes and leukocytes. Systemic immune responses are required for the resistance and clearance of *S.* Enteritidis infection. Currently available vaccines induce specific immune responses in birds, protecting them against pathogens. However, the development of a vaccine as a strategy to control *S.* Enteritidis infection in birds has some disadvantages. Vaccines interfere with the detection of *Salmonella* by bacteriological and serological methods. Other concerns with the live vaccines include the development of antimicrobial resistant and dissemination of *S*. Enteritidis in the environment (Baggesen, 2006). Therefore, a natural alternative that boosts immune responses in birds would be useful to combat pathogen infection. Previous studies have shown that seaweeds prime the immune system in mammals and birds (Choi et al., 2014). Additionally, beneficial gut microbes such as *Lactobacillus* sp. and *Bifidobacterium* sp. selectively ferment polysaccharides found in seaweeds (Hu et al., 2006). Red seaweeds have been shown to modulate the immune response and microbiota (Kulshreshtha et al., 2014). However, no studies have investigated the application of dietary seaweeds in birds challenged with a pathogen.

Several natural alternatives such as marine products, organic acids, microbiota enhancers, probiotics, prebiotics, and herbal products have been evaluated as feed additives in poultry (Venkitanarayanan et al., 2013). Among these, prebiotics have been shown to selectively increase the growth of beneficial microbes and inhibit pathogen colonization. Prebiotics such as oligosaccharides (mannan-oligosaccharides, galacto-oligosaccharides, and fructo-oligosaccharides) in poultry feed have been shown to enhance the immune system of birds (O’Sullivan et al., 2010). Among various health enhancing prebiotics, seaweeds have gained interest as potential feed additives in poultry (Richmond, 2004). Recent studies have shown that dietary inclusion of seaweed stimulates the health and productivity of birds by increasing the beneficial gut bacteria and enhancing the host innate immune system (Abudabos et al., 2013; Evans and Critchley, 2014).

In the present study, laying hens challenged with *S.* Enteritidis were used to determine the efficacy of the red seaweeds, *Chondrus crispus* (*C. crispus*) or *Sarcodiotheca gaudichaudii* (*S. gaudichaudii*). The effect of these two red seaweed species was investigated on the maintenance of body weight***(***BW), egg production, cecal microbiota, short chain fatty acids, and serum IgA production. A basal layer diet and antibiotic Chlortetracycline served as negative and positive controls, respectively.

## Materials and Methods

### Birds and Housing

A total of 96 commercial laying hens (Lohmann Lite) at 78 weeks of age were used in this study. Birds were randomly housed in 96 wire cages with *ad libitum* access to feed and water. Birds were confined in an environmentally controlled room set at 16 h of light per day and a temperature of 25°C. All experimental procedures were carried out in accordance with Canadian Council of Animal Care guidelines (CCAC, 2009) and University of Montreal Animal Care and Use Committee guidelines.

### Preparation of Seaweed Supplemented Feed and Experimental Design

Red seaweeds [*Chondrus crispus* (CC) and *Sarcodiotheca gaudichaudii* (SG)] used in this study were supplied by Acadian Seaplants Limited, Dartmouth, NS, Canada and Chlortetracycline (Aureomycin) was purchased from Alpharma Canada, Mississauga, ON. Two-dried seaweeds (SG and CC) were incorporated at 2 and 4% (w/w) of the total layer diet (Supplementary Table S1). A basal layer diet was used as the negative control and Chlortetracycline (110 ppm) was added to the basal layer diet to serve as the positive control. There were six dietary treatment combinations with 16 cages per treatment, and the experimental unit was a cage/bird.

### Layer Performance and Egg Quality

All eggs laid were collected daily and eggs produced per cage were recorded throughout the experiment. BWs (per bird per cage) and feed consumption were measured at the end of the weeks 1, 2, 3, and 4. Feed was weighed each day before being added to the feeders and feed consumption was calculated by re-weighing the feeders at the end of the time period. The data was used to calculate feed intake (FI) and egg production percentage (hen per day egg production).

### *Salmonella* Enteritidis Challenge

*Salmonella* Enteritidis field strain isolated from a clinical case of salmonellosis in laying hens was obtained from the Faculty of Veterinary Medicine collection center (SHY-04-1540, Chair in Poultry Research, University of Montreal). *S.* Enteritidis was cultured in LB broth overnight at 37°C, shaking at 150 rpm. To determine the number of colony-forming units, the inoculum was diluted and plated on XLD agar for 24 h at 37°C. During week-4 post feeding, one half of the laying hens were orally gavaged with 2 mL of the *Salmonella* culture suspended in PBS at a concentration of 10^9^ CFU/mL, and the non-challenged groups were mock inoculated with sterile PBS. This was done to mimic a natural horizontal infection in half of the hens. The infection protocol was based on results obtained from previous trials in the laboratory.

### Colonization of *Salmonella* Enteritidis in the Excreta Samples

On days 1, 3, 5, and 7 post challenge with *S.* Enteritidis, three discreet samples of bird excreta were collected with sterile spatula from all the cages. Excreta samples from two cages were pooled to represent one sample unit. Care was taken to avoid feed spillage and to prevent bird feathers from being picked up along with the excreta. Each collection was weighed and placed in zip lock bags. *S.* Enteritidis in the excreta samples was determined by blending 1 mL of the pooled sample into 10 mL of sterile solution of 0.1% peptone in water in a sterile bag. The obtained homogenates were serially diluted in peptone water and plated on XLD agar plates for the enumeration of *S.* Enteritidis. Plates were incubated for 24–48 h at 37°C and observed for the presence of *Salmonella* colonies, which appeared as black on the selective medium (Goodnough and Johnson, 1991). Suspected colonies were confirmed by biochemical tests and PCR analysis.

### Colonization of *Salmonella* Enteritidis in Egg Yolk

Eggs were collected daily, post challenge, to test for the presence of *S.* Enteritidis in the yolk. Each egg was broken aseptically and the yolk samples were placed in a sterile Nasco Whirl-Pak (WP) bag containing 50 mL of Hajna tetrathionate (HT) broth for 10 min (Miyamoto et al., 1997). The bags were homogenized and cultured at 37°C for 24 h. After incubation, a loop full of broth culture was spread onto a mannitol lysine crystal violet brilliant green (MLCB) agar (Nissui) plate to detect the presence of *S.* Enteritidis. After incubation for 24–48 h at 37°C, *Salmonella* colonies were detected as dark black center on the plates. Suspected colonies were confirmed by biochemical tests and PCR analysis.

### Colonization of *Salmonella* Enteritidis in Organs

All birds were euthanized by carbon dioxide gas inhalation and organs (ceca, spleen, liver, and ovary) were removed from each bird and placed in a sterile Nasco Whirl-Pak (WP) bag. Spleen and ovary samples were sent to Diagnostic Services, Faculty of Medicine, University of Montreal to test for the presence of *S.* Enteritidis. The *S.* Enteritidis count in the ceca was enumerated by the method described by Woodward et al. (2005) with modifications. Briefly, the contents of ceca were expelled by finger pressure into separate collection tubes and the empty segments were collected aseptically into pre weighed 15 mL sterile plastic tubes. The ceca segments were weighed and homogenized by diluting in peptone water to an initial 10^-1^ dilution. The homogenates in all treatments were serially diluted in peptone water and plated on XLD agar plates for the enumeration of *S.* Enteritidis. The suspected colonies were confirmed by biochemical tests and PCR analysis.

### Collection of Blood Samples and Biochemical Analysis

Blood samples were collected from the brachial vein on day 7 post *S.* Enteritidis challenge in Vacutainer tubes containing sodium heparin to determine the serum component (Lan et al., 2005). Approximately 8 mL of blood was collected from all the birds, using an 18-gauge needle. Blood sera was isolated by centrifuging the tubes at 3,000 × *g* for 10 min at 4°C. The separated serum, was transferred into individual vials and stored at -20°C for biochemical analysis. Whole blood samples were used to determine the WBC and RBC count. The whole blood and serum analysis was carried out at the Diagnostic Services, Faculty of Medicine, University of Montreal.

The serum immunoglobulin (IgA) were measured using a Chicken IgA ELISA kit (Bethyl Laboratories, Inc., Cedarlane, Hornby, ON, Canada). Serum IgA present in the sample was captured by anti-chicken IgA antibody pre-absorbed on the surface of microtiter wells. The quantity of IgA analyte present in the sample was proportional to the absorbance at 450 nm.

### Analysis of Intestinal Microbiota

The population of microbes within the gut was determined by quantitative, real time PCR using a protocol developed in our previous study (Kulshreshtha et al., 2014). Additionally, DNA samples were sent for next generation sequencing analysis to McGill University and Genome Quebec Innovation Centre, Montreal, Quebec.

### Gas Chromatographic Analysis of Cecal Contents

The short chain fatty acids (SCFA) in the cecal contents were analyzed as described by Martin et al. (2007). Briefly, 150 mg of cecal contents (*n* = 4) were homogenized in 1 ml of buffer, containing 0.1% (w/v) HgCl_2_, 1% (v/v) H_3_PO_4_ and 0.045 mg/ml 2,2 dimethyl butyric acid, as an internal standard. The diluted slurry was centrifuged at 500 × *g* for 30 min and the supernatant containing SCFA was collected for analysis (Martin et al., 2007). The sample (0.5 μL) was analyzed using a gas chromatograph (BRUKER 430), with a flame ionization detector (FID). A DB-FFAP (Dipheny- Free Fatty Acid Phase) column (Agilent Technology; length 30 m, internal diameter 530 μm with 1 μm film thickness) was used in the analysis. A cleaning injection of 1.2% formic acid wash was run following each sample run. The initial temperatures for each sample run for the oven, injector and detector were 80, 180, and 220°C, respectively. The SCFA in the samples were identified using an external standard containing acetate, propionate, *iso*-butyrate, *n*-butyrate, *iso*-valerate and *n*-valerate and quantified using an internal standard. The concentration of SCFA in the samples was determined as described by Zhao et al. (2006).

### Statistical Analysis

Pairwise comparisons were used to determine differences among treatment groups for incidences of *S.* Enteritidis colonization of the ceca, spleen, and ovary. The main effects of diets, bird age and the interaction between these effects were analyzed using ANOVA, significant difference with a *P*-value < 0.05 using the Proc. mixed procedure, of the SAS Institute, Inc. software version 9.3 (SAS Institute, Inc., Cary, NC, USA). When significant treatment effects were found, means were separated using Tukey analysis to differentiate treatment means.

## Results

### Red Seaweed Diets Maintained Body Weight and Laying Activity of Hens

The effects of red seaweed dietary supplements on FI, BW, and egg production are summarized in **Table 1**. Average FI and BW of each treatment group was similar at the beginning of trial but diverged (*P* < 0.0001) by the end of the trial. No interactions between the seaweed inclusion and bird age on response variables was detected until week-3 of the trial. However, there were significant treatment effects on BW and FI at week-4 upon inoculation of the birds with *Salmonella* Enteritidis (SE). Post challenge with *S.* Enteritidis, the seaweed supplemented (SG and CC) birds maintained higher (*P* < 0.0001) BW (1486–1526 g) than control birds (1245 g). Additionally, birds fed with Chlortetracycline, also had higher BW (1554 g) compared to the control. Similarly, the FI of birds fed seaweed diets was not significantly different from the control until week-3. At week-4, birds supplemented with seaweeds and Chlortetracycline showed an increase in FI (102.2–107.6 g/day) compared to the control birds (74.7 g/day). FI of birds fed Chlortetracycline was significantly higher in week-2 (*P* = 0.05), week-3 (*P* = 0.04), and week-4 (*P* = 0.01) compared to week-1.

**Table 1:** Effect of red seaweeds dietary supplements on the growth and performance of laying hens **(A)** Feed intake (FI) **(B)** Body weight (BW), and **(C)** Egg production.

Diets^1^	Weeks			
	1	2	3	4
**(A) Feed intake (g/d)**				
CC2	101.3ˆab	104.0ˆab	103.6ˆab	102.2ˆab
SG2	100.9ˆab	107.6ˆa	106.4ˆa	105.8ˆa
CC4	91.2ˆabc	104.0ˆab	106.7ˆa	107.6ˆa
SG4	87.2ˆabc	98.5ˆab	98.5ˆab	104.3ˆab
ANTB	84.0ˆbc	104.4ˆab	104.0ˆab	107.2ˆa
C	97.1ˆab	102.0ˆab	91.5ˆabc	74.7ˆc
SEM^2^	3.98			
**Interactions, *P*-value**				
Diets	<0.0001			
Weeks	0.005			
Weeks × Diets	<0.0001			
**(B) Body weight (g)**				
CC2	1532.4ˆab	1567.9ˆa	1572.3ˆa	1526.5ˆab
SG2	1565.7ˆa	1573.4ˆa	1570.5ˆa	1513.7ˆab
CC4	1561.8ˆa	1571.5ˆa	1544.7ˆab	1523.7ˆab
SG4	1553.0ˆab	1529.7ˆab	1529.7ˆab	1486.4ˆab
ANTB	1597.8ˆa	1630.0ˆa	1609.5ˆa	1554.0ˆab
C	1616.3ˆa	1566.1ˆa	1389^bc^	1245^c^
SEM^2^	32.63			
**Interactions, *P*-value**				
Diets	<0.0001			
Weeks	<0.0001			
Weeks × Diets	<0.0001			
**(C) Egg production (%)**				
CC2	75.5ˆabc	79.6ˆab	80.6ˆab	81.6ˆab
SG2	71.4ˆabc	86.7ˆa	82.7ˆab	91.8ˆa
CC4	50.9ˆbcd	66.3ˆabcd	68.4ˆabcd	71.4ˆabc
SG4	71.4ˆabc	72.4ˆabc	79.6ˆab	83.7ˆab
ANTB	44.9ˆcd	73.5ˆabc	79.6ˆab	80.6ˆab
C	75.5ˆabc	87.8ˆa	74.5ˆabc	35.7ˆd
SEM^2^	6.38			
**Interactions, *P*-value**				
Diets	0.001			
Weeks	0.0014			
Weeks × Diets	<0.0001			

*^1^Values are means for each treatment (Trt) group (CC2: contains 2% of *Chondrus crispus*; SG2: contains 2% of *Sarcodiotheca* variant; CC4: contains 4% of *Chondrus crispus*; SG4: contains 4% of *Sarcodiotheca* variant; C: Control; ANTB: Antibiotics (Chlortetracycline).*

*^2^Standard errors of the mean (14 replicates of 14 hens each per treatment) and different letters indicate statistically significant mean values (*P* < 0.0001).*

The data from eggs/cage were collected from week-1 and the production percentage was calculated. This served as a base line to compare the effects of treatments over the trial period. The base line egg production was significantly lower in the birds fed Chlortetracycline and CC4 (*P* = 0.005). Consequently, hen-day egg production increased from week-2 to week-4 for all the treatments except the negative control. A drop in egg production rate was observed from week-1 (75.5%) to week-4 (35.7%) in birds fed the basal layer diet (control). At week-4, egg production rate was significantly higher (*P* < 0.0001) for birds fed SG and CC (71.4–91.8%) compared to negative control birds (35.7%). Also, egg production percentage for the birds fed Chlortetracycline increased from week-1 (44.9%) to week-4 (80.6%) which was significantly higher than control (35.7 in week-4, respectively) (**Table 1**). The BW, FI and egg production of birds did not differ significantly between Chlortetracycline and seaweed treatments.

### Red Seaweeds Diet Reduces *Salmonella* Enteritidis Colonization in Excreta

All hens were clinically normal throughout the experiment and none of the excreta samples collected before the challenge were positive for *Salmonella* Enteritidis. The effect of dietary seaweed supplementation on the level of *S.* Enteritidis from excreta of birds is presented in **Table 2**. There were no significant differences among the treatments on day 1-Post Inoculation (PI). On day 3-PI, supplementation of diets with CC (2 and 4%) and Chlortetracycline reduced *S.* Enteritidis (*P* = 0.007) compared to control diets. However, SG supplementation (2 and 4%) was not different from control. Similar trends were observed on day 7-PI, where dietary inclusion of CC (2 and 4%) significantly reduced *S.* Enteritidis counts in excrement of birds compared to negative control birds. At day 5-PI, the colony counts of *S.* Enteritidis were significantly lower in the excreta samples of birds fed the higher inclusion level of CC (4%) and Chlortetracycline. However, recovery of *S.* Enteritidis from the excreta samples of birds fed SG (2 and 4%) and the lower inclusion level of CC (2%) were not significantly different from the control. No treatment effects were observed on day 1-PI on the recovery of *S.* Enteritidis from the excreta samples of birds (**Table 2**).

**Table 2:** The effect of red seaweed inclusion level on SE colonization (Log CFU/gram) in excreta of laying hens^1^.

Days	Diets^2^ (Log CFU/gram)						Interactions, *P*-value			
	CC2	SG2	CC4	SG4	Control	ANTB	SEM^3^	Diets	Days	Days × Diets
1	4.99^abcd^	5.53^ab^	4.53^abcde^	4.53^abcde^	5.14^abcd^	4.40^abcde^	0.20	<0.0001	<0.0001	0.001
3	3.28^efg^	4.12^bcde^	2.53^fg^	3.68^defg^	5.71^ab^	2.99^g^				
5	3.69^cdefg^	3.95^cdef^	2.29^g^	4.07^bcde^	5.21^abc^	2.29^g^				
7	3.24^efg^	4.08^bcde^	2.29^g^	4.06^bcde^	4.89^abcd^	2.29^g^				

*^1^Means represent eight excreta samples per treatment. Excreta were collected from 48 cages (1 hens/cage), sampling was done three times per cage and the total excreta samples from two cages were pooled to represent one sample unit.*

*^2^Values represented as log CFU/gram are means for each treatment (Trt) group (CC2: contains 2% of *Chondrus crispus*; SG2: contains 2% of *Sarcodiotheca* variant; CC4: contains 4% of *Chondrus crispus*; SG4: contains 4% of *Sarcodiotheca* variant; C: control; ANTB: antibiotics.*

*^3^Standard error of the mean (four replicates of eight hens each per treatment). Different letters indicate statistically significant mean values (*P* < 0.001).*

### Red Seaweed Diets Do Not Affect the Recovery of *Salmonella* Enteritidis from the Egg Yolk

No *S*. Enteritidis was recovered from the egg yolk samples from seaweed treatment or control birds on days 1, 3, 5, and 7-post inoculation.

### Red Seaweed Diets Reduce *Salmonella* Enteritidis Colonization of the Ceca in Laying Hens

**FIGURE 1:**
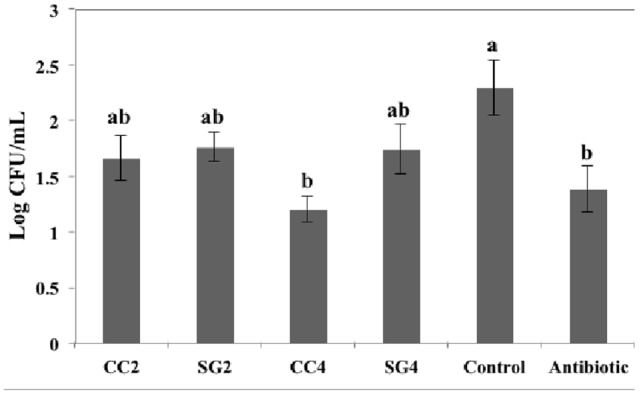
**Effect of red seaweed dietary supplements on the colonization of *S.* Enteritidis in the ceca of laying hens.** Data represent the mean of measurements from eight individual chicken ileum contents. Values with different superscript letters (Tukey multiple mean comparison) are significantly different (one-way ANOVA; *p* < 0.05). Values represent mean ± standard deviation (*n* = 8).

Colonization of *S.* Enteritidis in the ceca was determined at the end of the trial. The enumeration of *S.* Enteritidis revealed differences in their ability to colonize the ceca of infected birds. There was a significant reduction in the cecal *S.* Enteritidis count in birds fed seaweed diets compared to the control birds (**Figure 1**). Dietary supplementation of CC (4%) and Chlortetracycline significantly decreased (log CFU/gram = 1.19 and 1.3, respectively, *P* = 0.003) the bacterial colonization in the ceca compared to control (log CFU/gram = 2.29). No differences were observed in the average log CFU/g ceca among the birds supplemented with SG (2 and 4%), CC (2%) and control.

### Identification of *Salmonella* Enteritidis in Excreta and Cecal Samples

The isolated colonies (10/treatment) from the excreta and ceca samples were positively identified for *Salmonella* Enteritidis by the PCR method. Among the 96 birds used in the trial, two birds in the SG2 group showed feed withdrawal symptoms and were euthanized at the beginning of the trial. In all treatments (16 birds/treatment), *S.* Enteritidis was only recovered in the excreta and ceca of inoculated birds (eight birds/ treatment). This indicated that *S.* Enteritidis was not transmitted to the hens in the adjacent cages. Therefore, samples from infected hens only were tested for organ invasion, blood characteristics, microbiota and SFCA analysis; the results obtained showed consistent and uniform *Salmonella* colonization in the orally inoculated laying hens.

### Red Seaweed Diets Affect *S.* Enteritidis Colonization in the Organs

Recovery of *S.* Enteritidis from spleen and ovary samples of birds is shown in **Table 3**. Enrichment culturing of the spleen indicated higher frequencies (87–100%) of isolation of *S.* Enteritidis in hens fed 2 and 4% of SG compared to hens fed CC 2 and 4%, and those fed the basal layer diet (control, 62%). Recovery of *S.* Enteritidis in the spleen was lowest (37%) in the hens fed Chlortetracycline. However, the frequencies of *S.* Enteritidis recovery from ovaries did not differ significantly among the treatment groups except for SG4 (37%). Cecal samples isolated from all the birds fed SG4 were positive for *S.* Enteritidis. Control and SG2 had 87.5% *S.* Enteritidis positive ceca samples and CC4 and Chlortetracycline had the lower incidences (50%) of *S.* Enteritidis in the ceca (**Table 3**).

**Table 3:** Effect of red seaweed dietary supplements on the colonization of *S.* Enteritidis in the organs of laying hens^1^.

Diet^2^	*Salmonella* Enteritidis-positive hens/total hens (*n* = 8)		
	Ovary	Spleen	Ceca
CC2	0%	62.5%	75%
SG2	0%	100%	87.5%
CC4	12.5%	62.5%	50%
SG4	37.5%	87.5%	100%
C	0%	62.5%	87.5%
ANTB	0%	37.5%	50%

*^1^Values represented as percentage refers to the ratio of number of organs infected to the total number of organs.*

*^2^Values are means for each treatment (Trt) group (CC2: contains 2% of *Chondrus crispus*; SG2: contains 2% of *Sarcodiotheca* variant; CC4: contains 4% of *Chondrus crispus*; SG4: contains 4% of *Sarcodiotheca* variant; C: control; ANTB: antibiotics (Chlortetracycline).*

### Red Seaweed Diets Affect Blood Serum Profile of *S.* Enteritidis Infected Hens

The effect of red seaweeds on red blood cells, white blood cells counts and IgA are summarized in **Table 4**. Serum IgA concentration was increased (*P* < 0.001) for CC4 birds (19.83 mg/mL) compared to controls (12.50 mg/mL). SG2 treatment (33 × 10^9^/L) showed higher (*P* = 0.005) WBC counts than the control treatment (20.71 × 10^9^/L). There was no significant effect due to feed supplementation of seaweed on RBC count and blood serum concentrations of sodium, aspartate aminotransferase (AST) and albumin levels (**Table 4**).

**Table 4:** Effect of red seaweed dietary inclusion levels on blood cell counts and immunoglobulin concentration in laying hens infected with *S.* Enteritidis.

Diet^1^	IgA (mg/ml)	WBC^3^ (×10^9^/L)	RBC^4^ %	AST (IU/lit)	Alb g/L	Sodium (mmol/L)
CC2	16.68ˆab	16.41ˆc	28.00ˆa	224.57ˆa	19.23ˆab	145.23ˆa
SG2	14.98ˆb	33.00ˆa	27.33ˆa	185.71ˆa	20.46ˆa	144.20ˆa
CC4	19.83ˆa	26.29ˆab	28.57ˆa	171.43ˆa	19.10ˆab	145.21ˆa
SG4	10.47ˆc	18.81ˆc	26.17ˆa	235.29ˆa	16.13ˆb	139.89ˆa
ANTB	16.72ˆab	12.69ˆc	27.88ˆa	215.43ˆa	17.04ˆab	148.37ˆa
C	12.50ˆbc	20.71ˆbc	29.00ˆa	188.14ˆa	19.14ˆab	144.63ˆa
SEM^2^	2.87	7.82	2.83	108.53	2.19	6.59
*P-*value	<0.001	0.005	0.51	0.8	0.008	0.3

*^1^Values are means for each treatment (Trt) group (CC2: contains 2% of *Chondrus crispus*; SG2: contains 2% of *Sarcodiotheca* variant; CC4: contains 4% of *Chondrus crispus*; SG4: contains 4% of *Sarcodiotheca* variant; C: control; ANTB: antibiotics.*

*^2^Standard errors of the mean (eight replicates per treatment) and different letters indicate statistically significant mean values (*P* < 0.001).*

*^3^White blood cells.*

*^4^Red blood cells.*

### Red Seaweed Diets Alter Microbial Population in Ceca

**FIGURE 2:**
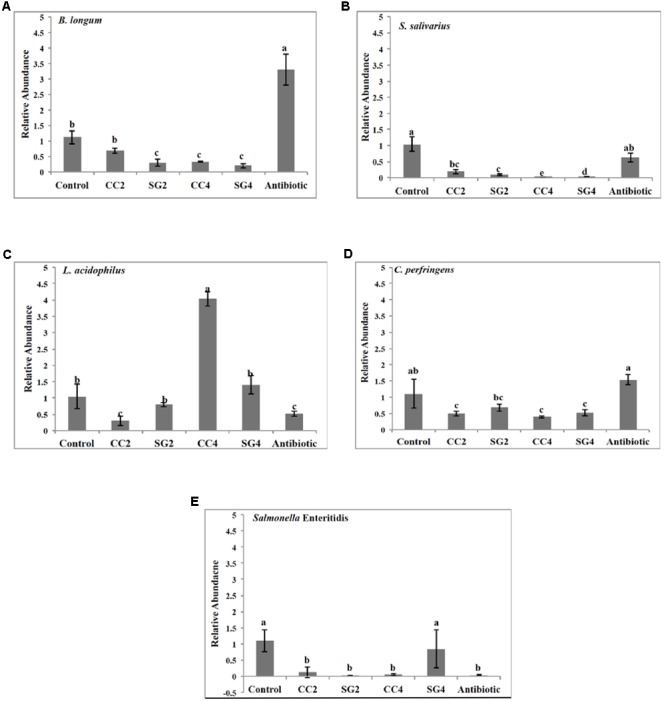
**Real time PCR quantification of microbial population in the cecal contents of laying hens.** Relative abundance of **(A)** *Bifidobacterium longum*, **(B)** *Streptococcus salivarius* **(C)** *Lactobacillus acidophilus*, **(D)** *Clostridium perfringens* **(E)** *S.* Enteritidis in the chicken ileum. Data represent the mean of measurements from two individual chicken ileum contents, Error bars indicate standard error and different letters indicate statistically significant mean values (*P* < 0.001).

**FIGURE 3:**
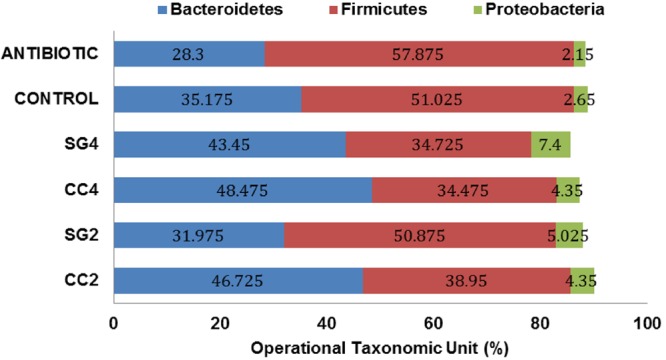
**Effect of red seaweed diets on the composition of cecal microbiota.** Values are means for each treatment (Trt) group (C: control; ANTIBIOTIC: Chlortetracycline; SG2: contains 2% of Sarcodiotheca variant; SG4: contains 4% of Sarcodiotheca variant; CC2: contains 2% of Chondrus crispus. CC4: contains 4% of Chondrus crispus Means represent four excreta samples per treatment (n = 4).

Red seaweed dietary inclusion altered the relative abundance of beneficial bacteria; *Bifidobacterium longum, Lactobacillus acidophilus, Streptococcus salivarius* and pathogenic bacteria, *Clostridium perfringens* and *Salmonella* Enteritidis in the ceca (**Figure 2**). The abundance of *L. acidophilus* increased (*P* < 0.001) by threefold in 4% CC treatments (**Figure 2C**). Additionally, the relative abundance of *B. longum* was twofold higher (*P* < 0.001) in the Chlortetracycline diet (**Figure 2A**). The relative abundance of *S. salivarius* was significantly lower in 2 and 4% CC and SG dietary supplementations (**Figure 2B**). Interestingly, all seaweed treatments except SG2 had a decreased in the prevalence of *C. perfringens* compared to the negative control (P < 0.001) (**Figure 2D**). The abundance of *S.* Enteritidis was significantly reduced in the CC 2 and 4% and Chlortetracycline treatments (**Figure 2E**). The sequence analysis showed that Firmicutes, Bacteroidetes, and Proteobacteria were the most dominant phylum in the ceca for all dietary treatments. These three predominant phyla in the ceca were significantly affected by the dietary treatments (**Figure 3**). The abundance of Bacteriodetes were found to be significantly reduced in the antibiotic treated group (26.67%, *P* = 0.007) compared to the control groups (34.75%). This reduction was not observed in seaweed treatment groups, where dietary inclusion of *C. crispus* (46.6–48.4%, *P* < 0.05) significantly increased Bacteriodetes compared to the control group (*P* < 0.0001). However, Firmicutes were predominant in the antibiotic treated group (57.87%) compared to the control (51.02, *P* = 0.0045) and the seaweed (CC, 34–38&, SG 34–50%, *P* < 0.05) treated groups. *C. crispus* and antibiotic dietary inclusion did not affect the relative abundance of Proteobacteria (*P* = 0.08 and 0.95, respectively; **Figure 3**).

### Red Seaweed Diets Alter Short Chain Fatty Acid (SCFA) Composition in the Digesta

Dietary inclusion with red seaweeds affected SCFA concentration in the digesta (**Table 5**). The concentration of propionic acid was higher (*P* = 0.003) in the cecal digesta of birds fed 4% CC (32.64), as compared with the basal diets (13.67 mmol/kg) or Chlortetracycline (17.60 mmol/kg). The concentration of *n-*butyric acid was higher in the Chlortetracycline treatment (*P* = 0.002) than the control. No significant differences in the concentration of acetic acid, *i*-butyric acid, *n*-valeric, and *i*-valeric acids were observed in any of the treatments (**Table 5**).

**Table 5:** Effect of red seaweed dietary supplements on the concentration of short chain fatty acids (mmol/kg) in the ceca digesta of laying hens infected with *S.* Enteritidis.

Diet^1^	Short chain fatty acids (mmol/kg)				
	Acetic acid	Propionic acid	*i*-Butyric acid	*n*-Butyric acid	*i*-Valeric acid
CC2	31.04ˆab	13.46ˆb	0.89ˆa	7.04ˆbc	0.76ˆa
SG2	23.03ˆb	8.49ˆb	0.31ˆa	3.05ˆc	0.29ˆa
CC4	70.58ˆa	32.64ˆa	0.58ˆa	8.37ˆbc	0.40ˆa
SG4	63.54ˆa	25.84ˆab	0.83ˆa	11.43ˆab	0.74ˆa
ANTB	44.50ˆab	17.60ˆab	0.84ˆa	17.39ˆa	1.06ˆa
C	33.01ˆab	13.67ˆb	0.50ˆa	5.93ˆbc	0.50ˆa
SEM^2^	17.7	7.72	0.38	3.38	0.34
*P-*value	0.007	0.003	0.25	0.002	0.05

*^1^Values are means for each treatment (Trt) group (CC2: contains 2% of *Chondrus crispus*; SG2: contains 2% of *Sarcodiotheca* variant; CC4: contains 4% of *Chondrus crispus*; SG4: contains 4% of *Sarcodiotheca* variant; C: control; ANTB: antibiotics (Chlortetracycline).*

*^2^Standard errors of the mean (four replicates per treatment) and different letters indicate statistically significant mean values (*P* < 0.001). Means represent four ceca digesta samples per treatment.*

## Discussion

In spite of significant progress in the improvement of food safety by implementation of several pathogen control strategies on-farm and in poultry processing units, the incidences of *Salmonella* infection in humans have remained relatively unchanged with an estimated 500 to 2000 deaths each year (WHO Global *Salmonella* survey program; Betancor et al., 2010; Yim et al., 2010). In addition to the on-farm intervention strategies, an alternate step to decrease *S.* Enteritidis colonization in birds is critical to reduce the incidence of associated human illness (Rajic et al., 2007). As *S.* Enteritidis is zoonotic and has shown increasingly high resistance to various antimicrobials, a natural feed additive such as seaweeds could serve as a better alternative.

In the present study, we quantified the effect of dietary inclusion of red seaweeds on the prevalence of *Salmonella* in laying hens. Supplementation of layer feed with red seaweeds, *Chondrus crispus* and *Sarcodiotheca gaudichaudii*, resulted in a significant reduction of *S.* Enteritidis in the ceca, maintained BW and egg production and increased the concentration of blood serum immunoglobulin (IgA) and the population of beneficial bacteria in the ceca.

To replicate the conditions of *S.* Enteritidis infection in a commercial poultry farm, half of the total number of the birds/treatment (8/16 birds per treatment) were challenged with *S.* Enteritidis and the remaining birds were expected to develop infection by horizontal transmission of bacteria. Surprisingly, *S.* Enteritidis was not recovered from any of the non-challenged hens in the trial. This could be either due to lower level of bacteria shed into the environment or due to reduced colonization of *S.* Enteritidis in the challenged hens. The possible mechanisms of horizontal transmission of *S.* Enteritidis in birds could be by contaminated feed, water supply, feathers and excrement or due to the aerosolized *S.* Enteritidis released into the environment (Bailey et al., 1990; Baskerville et al., 1992). In the present study, *S.* Enteritidis colonized and persisted in the cecum of challenged hens (**Figure 1**), however, the lower level of bacteria shed into the environment could likely be responsible for the absence of *S.* Enteritidis in the cecal and fecal samples isolated from non-challenged birds. Asakura et al. (2001) demonstrated that housing birds at higher stock density and in unsanitary conditions increases their susceptibility to *S.* Enteritidis infection (Asakura et al., 2001). Additionally, in our study, a low stress level in the room (1 bird/cage), regular changing of feed troughs and frequent cleaning of manure trays post-inoculation might have lowered the bacterial shedding into the environment.

In the present study, the addition of red seaweeds in the feed did not affect FI, BW and egg production of the birds until week-3 of the trial. However, the FI, BW and egg production in the control birds declined significantly from week-4 until the end of the trial (**Table 1**). The drop in BW and egg production coincided with the time that the birds were inoculated with *S.* Enteritidis. Thus, it can be inferred that the *S.* Enteritidis challenge depressed the growth parameters and egg production rate in control birds. This could be due to the anorexic response of birds to infection. Additionally, in control birds, the available energy would most likely be reallocated toward immune development to combat *S.* Enteritidis infection. This would have caused inefficient nutrient utilization for growth and egg production, thus resulting in detrimental effects on the health (BW) and productivity (egg production) of birds (Cox et al., 2010). However, the Chlortetracycline and seaweed dietary supplementation maintained FI, egg production and reduced BW loss due to *S.* Enteritidis infection (**Table 1**). This could be due to the immune enhancing effect of red seaweeds. The role of oligosaccharide-rich diet, like seaweeds, in animal feed has been well researched. Dietary inclusion of seaweeds has been shown to improve growth performance and gut microbiota of birds by enhancing the immune system (Strand et al., 1998). Likewise, in the present study, supplementation of layer feed with two red seaweeds (CC and SG) maintain FI and BW most likely by improving immune status, thus resulting in sufficient nutrient availability for advancing growth. Additionally, the egg production rate in week-1 was significantly lower in CC4 and Chlortetracycline supplemented birds (**Table 1**). The lower egg production in week-1 could be due to the birds’ response to the transportation during the start of the trial. A drop in egg production may indicate a stress induced coping strategy related to the transport (Braastad and Katle, 1989).

Upon consumption of *S.* Enteritidis contaminated water or feed, *S.* Enteritidis enters the hens’ esophagus and colonizes intestinal cells, passes through the mesenteric lymph nodes, and is carried by macrophages to spread in the organs (Okamura et al., 2001). In our study, *S.* Enteritidis fecal shedding was significantly reduced in seaweed (CC) supplemented birds compared to controls (**Table 2**). Several reports have indicated that cell mediated immunity is responsible for the clearance of *S.* Enteritidis from the tissue, while humoral immunity is critical for the reduction of intestinal colonization (Babu et al., 2004; Van Immerseel et al., 2005). Previously, sulphated polysaccharides obtained from an edible fungus *Agrocybe chaxingu* significantly augmented the level of both cellular and humoral immunity in broiler chicks (Zhang et al., 2013). Moreover, sulphated polysaccharides from marine sources have been shown to enhance the activity of neutrophils and, macrophages, and to function as an activator of the humoral immune response (Kuznetsova et al., 2003). Similarly, in the present study, sulphated polysaccharides of red seaweed possibly induced the immune response of the birds resulting in the reduced fecal shedding of SE. The primary site for *S.* Enteritidis colonization is cecum and then infection spreads to the spleen and liver by lymphatic routes. The number of *S.* Enteritidis positive samples was highest in the ceca, followed by the spleen and lowest in the reproductive tract, consistent with the previous reports (Gantois et al., 2008; Cho et al., 2010). *Chondrus crispus* supplemented feed was effective in reducing *S.* Enteritidis count in the excreta and contents of the ceca. This could be due to the ability of seaweed to block the initial attachment of bacteria to the epithelial cells by targeting the motility and virulence of *S.* Enteritidis (Kulshreshtha et al., 2016). Furthermore, none of the egg yolk samples were positive for *S.* Enteritidis in the trial. This is in agreement with the previous research by Garcia et al. (2011), where no *Salmonella* spp. was detected in the egg content of *S.* Enteritidis infected birds. This could be due to the protective effect of egg’s complex system of antimicrobial components (Garcia et al., 2011).

Although, the exact mechanism (s) of action of seaweed dietary supplements on pathogens or the health of the chicken is unclear, it has been suggested that the pathogen inhibitory effect can be correlated with metabolic characteristics of health enhancing bacteria and their immunomodulation activity. Probiotic bacteria such as *Lactobacillus* have been shown to suppress the growth of pathogens by mechanisms such as competitive exclusion, secretion of SCFA, and antimicrobial peptides as well by priming the host immune system (Torok et al., 2008). The population of probiotics such as *Lactobacillus* can be altered by dietary inclusion of prebiotics, which selectively enhance their growth. Interestingly in the present study, a negative correlation was observed between the relative abundance of *Lactobacillus acidophilus* and *Salmonella* Enteritidis in the birds supplemented with CC (4%) (**Figure 3**), suggesting *Lactobacillus acidophilus* as a suppressor of *Salmonella* Enteritidis (Gibson et al., 2005). Moreover, this could be due to the effect of dietary *C. crispus* on the epithelial cell turnover, thus altering the growth in favor of the beneficial bacteria (Kulshreshtha et al., 2014). Previously, it has been documented that the host microbiota composition is dependent on the dietary carbohydrate intake (Rinttilä and Apajalahti, 2013). Supplementing polysaccharides rich seaweeds, CC and SG altered the cecal microbiota of laying hens after *S.* Enteritidis infection. Significant changes in the abundance of genera belonging to phylum *Firmicutes* and *Bacteroidetes* were observed with *C. crispus* (4%) supplementation, representing the treatment group showing the maximum protection against colonization of SE. This indicates that dietary supplementation of *C. crispus* to layer hens altered their microbiota composition, and imparted resistance against *Salmonella*. An increase in *Bacteroides* was observed with CC dietary inclusion. In the chickens’ ceca, genus *Bacteroides* are one of the most dominant anaerobic genera that play an important role in the breakdown of complex molecules such as polysaccharides into simpler compounds to provide energy (Reeves et al., 1997). The increase in the abundance of *Bacteriodes* could be due to the bioavailability of undigested fermentable polysaccharides as a result of *C. crispus* supplementation. Thus, the selectivity of fermentation substrates could most likely be the reason for the shift in the cecal microbiota with the *C. crispus* supplementation. Short-chain fatty acids are fermentation end products of gut microbes, which leads to the proliferation of colonocytes and they also have inhibitory effects on the growth of pathogens such as *Salmonella* (Hinton et al., 1990). Previously, dietary inclusion of seaweeds has been shown to increase the cecal concentration of acetate and propionate (Gomez-Ordonez et al., 2012). The intestinal fatty acid propionate, controls *Salmonella* invasion through post-translational modification of HilD, which is a major regulator of *Salmonella* pathogenicity island 1 (SPI-1) (Hung et al., 2013). Thus, an increase in propionate concentration in birds fed on seaweed (CC, **Table 5**) would have repressed the SPI-1 regulator (HilD), leading to decreased expression of effector proteins, eventually resulting in decreased *S.* Enteritidis penetration into the cells.

Probiotic bacteria such as *Lactobacillus* can modulate immune response by stimulating the production of immunoglobulin such as IgA. Immunoglobulin prevents the pathogen colonization by blocking its attachment to the epithelial cell receptors (Rocha et al., 2012). Therefore, in the present study, an increase in the production of IgA could be correlated to the reduced colonization of *S.* Enteritidis. Moreover, higher levels of *Lactobacillus* could have resulted in higher IgA production.

## Conclusion

Red seaweed dietary supplementation of layer feed reduced the negative effect on layer growth and egg production caused by *Salmonella* Enteritidis. Dietary inclusion of *Chondrus crispus* (CC) inhibited colonization of *Salmonella* in the excreta and ceca. This could be by promoting the growth of *Lactobacillus* and increasing the concentration of SCFAs. A higher level of IgA in birds supplemented with CC indicates a direct role of seaweed on the maturation of the humoral immune system. Since chickens are a major reservoir of *S*. Enteritidis, innovative on-farm strategies such as seaweed feed supplements for reducing *S.* Enteritidis colonization in birds can serve as an effective alternative to controlling human infections, transferred from chicken to human. Additionally, producers (including organic farmers), will likely readily accept a natural feed additive like seaweed, which is non-toxic.

## Author Contributions

BP, BR, MB, GS, NT, and GK conceived and designed the experiments. GK, LB, and JM performed experiments. GK, BP, and BR analyzed and interpreted the data. AC and JH contributed reagents, materials, and analysis tools; and GK, BP, BR, and GS wrote the paper.

## Conflict of Interest Statement

The authors declare that the research was conducted in the absence of any commercial or financial relationships that could be construed as a potential conflict of interest.
