# Supplementary material for: Feed Supplementation with Red Seaweeds, Chondrus crispus and Sarcodiotheca gaudichaudii, Reduce Salmonella Enteritidis in Laying Hens
Source: Front Microbiol. 2017 Apr 10;8:567. doi: 10.3389/fmicb.2017.00567 (PMC5385333; doi:10.3389/fmicb.2017.00567)
Supplement: Supplementary file 1 [file Table_1.PDF]

Table S1. Dietary composition of experimental diets containing seaweeds

Ingredients % as feed	Seaweed (%)[1]					
	C	ANTB	CC2	CC4	SG2	SG4
Corn	50.61	50.59	48.46	46.11	48.31	46.02
Soybean meal	24.13	24.13	23.68	23.26	23.72	23.31
Wheat	10.00	10.00	10.00	10.00	10.00	10.00
SG Meal	-	-	-	-	2.0	4.0
CC Meal	-	-	2.0	4.0		-
Ground Limestone	5.04	5.04	5.04	5.04	5.04	5.04
Mono-Dicalcium Phosphorus	1.17	1.17	1.16	1.16	1.17	1.16
Oyster shell	2.52	2.52	2.52	2.52	2.52	2.52
Shell mix	2.52	2.52	2.52	2.52	2.52	2.52
Poultry fat	2.72	2.72	3.53	4.42	3.58	4.45
Antibiotic	-	0.004	-	-	-	-
MCL4[2]	0.50	0.50	0.50	0.50	0.50	0.50
Methionine Premix[3]	0.45	0.46	0.46	0.47	0.46	0.47
Iodized salt	0.34	0.34	0.11	0.00	0.18	0.01
Total	100	100	100	100	100	100
Nutrient content, Calculated values						
Metabolizable energy (kcal-kg^{-1})	2850.03	2850.03	2850.03	2850.03	2850.03	2850.03
Protein (%)	15.59	15.59	15.59	15.59	15.59	15.59
Crude fiber (%)	2.28	2.28	2.28	2.28	2.28	2.28
Calcium (%)	4.17	4.17	4.17	4.17	4.17	4.17
Non-Phytate phosphorus (%)	0.35	0.35	0.35	0.35	0.35	0.35
Lysine (%)	0.82	0.82	0.82	0.82	0.82	0.82
Methionine + cysteine (%)	0.68	0.68	0.68	0.68	0.68	0.68

[1]Treatment group, CC2: contains 2% of *Chondrus crispus* ; SG2: contains 2% of *Sarcodiotheca* variant; CC4: contains 4% of *Chondrus crispus*; SG4: contains 4% of *Sarcodiotheca* variant; C: Control; ANTB: Antibiotics (Chlortetracycline)

[2]Providing per kg of diet: retinol, 7,800 IU/kg; cholecalciferol, 2,500 IU/kg ; DL-alpha-tocopherol acetate, 20 IU/kg;; thiamine, 1.94 mg/kg; riboflavin, 7.6 mg/kg; pantothenic acid, 7.2 mg/kg; niacin, 30.7 mg/kg; pyridoxine, 3.96 mg/kg; choline chloride, 641mg/kg ; vitamin K, 2.97 mg/kg; biotin 0.16 mg/kg; cyanocobalamin, 12 mg/kg; antioxidant, 1 mg; manganese, 70.2 mg/kg; zinc, 66 mg; iron, 33 mg; copper sulphate, 25 mg/kg; iodine, 0_9 mg; selenium, 0.15 mg/kg ethoxyquin, 50 mg/kg; folic acid, 0.66 mg/kg.

[3]Methionine premix is composed of 50% wheat middlings and 50% DL methionine.
